# One‐Pot RPA‐CRISPR/Cas12a Assay With Visual Readout for the Ultra‐Specific Detection of Monkeypox Virus Clade I

**DOI:** 10.1111/1751-7915.70437

**Published:** 2026-09-06

**Authors:** Boyi Li, Luyao Liu, Kaikai Jin, Zanheng Huang, Tianyi Zhang, Rong Gao, Huanxin Chen, Lijuan Niu, Changqi Fan, Haili Zhang, Pei Huang, Hualei Wang

**Affiliations:** ^1^ State Key Laboratory for Diagnosis and Treatment of Severe Zoonotic Infectious Diseases, Key Laboratory for Zoonosis Research of the Ministry of Education, Institute of Zoonosis, College of Veterinary Medicine Jilin University Jilin China; ^2^ Zhili College, Tsinghua University Beijing China

**Keywords:** CRISPR/Cas12a, MPXV clade I, one‐pot detection, RPA

## Abstract

In 2024, Monkeypox virus (MPXV) clade I has triggered outbreaks in several countries world‐wide. MPXV clade I demonstrates enhanced virulence and transmissibility, with a case fatality rate reaching 10%. In response, we have developed a one‐pot detection assay specifically targeting MPXV clade I, combining recombinase polymerase amplification (RPA) and the CRISPR/Cas12a system. The assay can be completed within 40 min and achieved a 95% limit of detection (LOD95) of 27.16 copies/μL. No cross‐reactivity was observed with MPXV clade II or other tested viral templates, including Vaccinia virus (Tiantan strain). A preliminary room‐temperature evaluation showed that the assay retained detectable performance at 25°C, supporting its potential use in equipment‐limited settings. The assay also showed good intra‐assay and inter‐assay repeatability for recombinant plasmid templates, with all coefficient of variation (CV) values below 10%. In simulated clinical samples, the RPA‐CRISPR/Cas12a assay detected more low‐concentration plasmid‐spiked samples than quantitative polymerase chain reaction (qPCR). These results indicate that the established assay is specific, sensitive, repeatable, and easy to perform, providing a practical tool for field‐based screening and decentralized detection of MPXV clade I.

## Graphical abstract

This study established a rapid RPA‐CRISPR/Cas12a assay for MPXV clade I detection, achieving an LOD95 of 27.16 copies/μL within 40 min. The assay exhibited no cross‐reactivity with MPXV clade II or other orthopoxviruses. Under simulated conditions, the assay detected more low‐concentration plasmid‐spiked samples than quantitative polymerase chain reaction (qPCR).

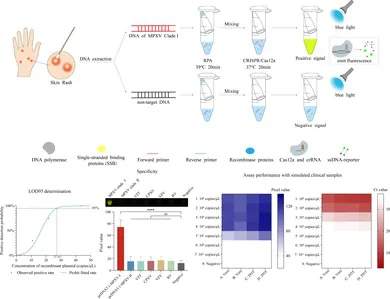

## 1 Introduction

Monkeypox virus (MPXV), a double‐stranded DNA virus of the *Orthopoxvirus* genus in the family Poxviridae, serves as the causative pathogen of Mpox. The viral species is segregated into two principal genetic clades: clade I and clade II (Amir et al. 2025; Halder et al. 2025; He et al. 2025). Since 2022, Mpox outbreaks have been emerging globally, and the emerging sublineage Ib of MPXV clade I has triggered further outbreaks in several countries world‐wide in 2024 (Bragazzi et al. 2022; Du et al. 2025). Risk of transmission of MPXV clade Ib extends beyond sexual contact, with confirmed household transmission through close contact, posing significant threats to paediatric populations (Mukadi‐Bamuleka et al. 2024; Srivastava et al. 2024). Children below the age of 15 comprise about 70% of confirmed cases in the current Democratic Republic of the Congo outbreak, while representing a striking 88% of fatalities documented to date (Srivastava et al. 2024).

Distinct from clade II, MPXV clade I is associated with increased virulence, driving higher mortality and more severe disease outcomes (Luna et al. 2022; Americo et al. 2023; McGrail et al. 2024). This severity necessitates closer clinical oversight and earlier therapeutic escalation (Lee et al. 2024). Compounding this clinical burden is clade I's enhanced transmissibility, which underscores the necessity for proactive, real‐time genomic surveillance to interrupt chains of transmission (Okwor et al. 2023). In this setting, the capacity for rapid clade discrimination transcends basic technical utility, representing a fundamental requirement for rigorous epidemiological inquiry and the implementation of precise containment protocols. Currently, PCR retains its status as the WHO‐endorsed reference assay for MPXV detection (World Health Organization 2024). But PCR has limitations, including prolonged turnaround time and dependence on expensive and technically demanding thermal cyclers (Liu and Yang 2025). The development of a rapid point‐of‐care detection assay specifically targeting MPXV clade I is therefore urgently required.

**FIGURE 1:**
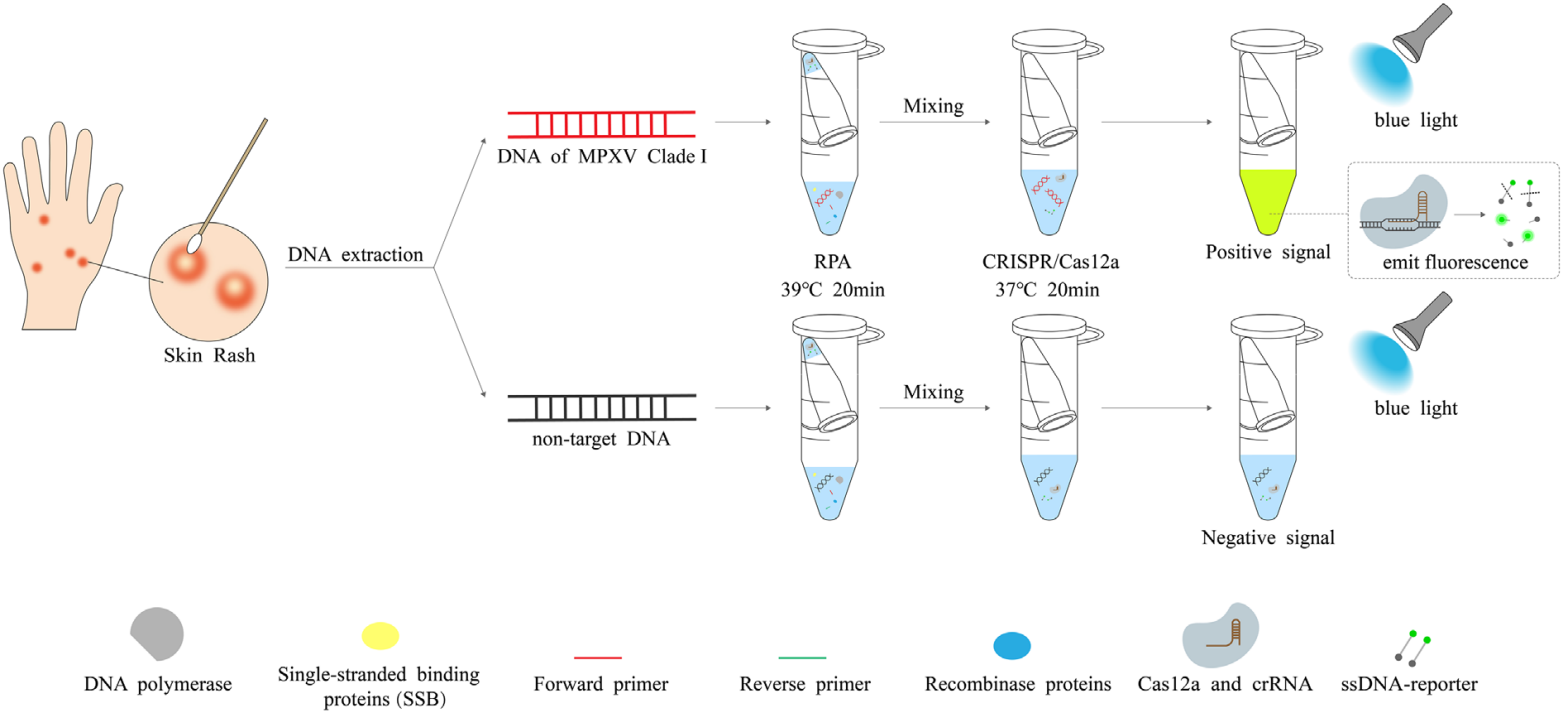
Schematic diagram of the RPA‐CRISPR/Cas12a assay. The assay was performed using a compartmentalized tube sleeve consisting of a 1.5 mL microcentrifuge tube and an inverted capless PCR tube. Extracted nucleic acids were added to the RPA reaction mixture at the bottom of the 1.5 mL tube, while the CRISPR/Cas12a detection mixture was retained in the inverted PCR tube by surface tension. After RPA amplification at 39°C for 20 min, brief centrifugation released the CRISPR/Cas12a mixture into the amplified RPA reaction. The combined reaction was then incubated at 37°C for 20 min, and the results were visualized using handheld blue‐light transillumination or smartphone‐based image documentation.

The CRISPR/Cas system, originally identified as an adaptive immune mechanism in bacteria and archaea, is now extensively utilized for viral pathogen detection (Li et al. 2022, 2023). The CRISPR/Cas system has demonstrated exceptional performance, although its sensitivity can still be enhanced through integration with amplification techniques (Liu et al. 2022). Therefore, isothermal amplification assays such as Recombinase Polymerase Amplification (RPA) have been used in combination with CRISPR/Cas (Mao et al. 2023). RPA is a highly sensitive and specific isothermal amplification technique that operates at 37°C–42°C and achieves exponential amplification within a short time frame (Tan et al. 2022). The integrated RPA‐CRISPR/Cas12a system has been widely applied in nucleic acid detection due to its advantages of simplicity, rapidity, and high efficiency (Li et al. 2023). In this study, we established an MPXV clade I detection assay based on the RPA‐CRISPR/Cas12a system (Figure 1), resulting in a rapid, accurate, and sensitive assay that does not require sophisticated equipment. This approach has potential for home‐based testing, and should provide technical support for MPXV clinical diagnosis and epidemiological investigations.

## 2 Materials and Methods

### 2.1 Preparation and Activity Validation of Cas12a Protein

The laboratory‐constructed expression plasmid pET28a‐LbCas12a containing Cas12a protein was transformed into Rosetta (DE3) competent cells. Single colonies were then selected and cells were subjected to expansion culture. We harvested the soluble fraction by sonicating a portion of the bacterial culture, followed by centrifugation. This cleared lysate was then batch‐bound to Ni‐NTA resin overnight at 4°C. After discarding the flow‐through and washing away nonspecific binders, we eluted the target protein and concentrated it using centrifugal ultrafiltration devices. Samples including the eluate, flow‐through, and purified protein were analysed with SDS‐PAGE using Coomassie Brilliant Blue staining to assess purification efficiency. The protein activity was validated using the laboratory‐established CRISPR/Cas12a assay.

### 2.2 Viral Nucleic Acids and Plasmids

Using SnapGene 6.0.2, we analysed MPXV genomes from diverse global and temporal isolates to identify clade‐specific conserved regions (Clade I: NC_003310.1, 48,077–48,897; Clade II: NC_063383.1, 46,051–46,868). These fragments were subcloned into pcDNA3.1, yielding pcDNA3.1‐MPXV‐I and ‐II. As outgroup controls, synthetic plasmids for Monkey B virus (BV) Gb and Cowpox virus (CPXV; NC_003663.2, 64,872–65,123 bp) were procured from General Biosystems (Anhui). Additionally, nucleic acids from Vaccinia virus (Tiantan strain, VVT) and Varicella‐zoster virus (VZV) were purified using a DNA extraction kit (BioPerfectus China).

### 2.3 Cell Culture

Experiments were carried out using the HEK 293 T cell line (RRID:CVCL_0063), sourced from Shanghai Huiying Biotechnology. To ensure a clean working stock, we treated the cells with a mycoplasma elimination reagent for over 2 weeks. This precaution paid off: the cells maintained their expected growth rate and passage frequency, indicating a healthy, contamination‐free culture.

### 2.4 Design of CRISPR RNA (crRNA) and RPA Primers

Based on the identification of PAM‐containing protospacer motifs within conserved regions of MPXV clade I, multiple template strands for crRNA transcription were designed and synthesized in accordance with the CRISPR/Cas12a ribonucleoprotein complex assembly principles. Subsequently, three pairs of RPA primers specific to the target sequences were designed using the Primer Premier 5.0 software. The template strands for crRNA transcription and RPA primers are listed in Table 1 and were synthesized by Sangon Biotech (Shanghai) Co. Ltd.

**TABLE 1:** Oligonucleotide sequences used in this study.

Name	Sequence (5–3′)
RPA‐1‐F	TCAAAAGACTTATGATCCTCTCTCATTGATTT
RPA‐1‐R	AGATCAGTTTTATTGCTAGTTGCGTTAGTTCT
RPA‐2‐F	GATCATACAGAGCTTTATTAACTTCTCGCTTC
RPA‐2‐R	AGATTCTTCCAGATAATAATCCATCTCCTCCA
RPA‐3‐F	CATAGCACTACGTTGAAGATCATACAGAGCTT
RPA‐3‐R	GAACGACGAACCACCAGAGGATGATGAATA
crRNA‐1	GAAAUUAAUACGACUCACUAUAGGGUAAUUUCUACUAAGUGUAGAUGUUAGUUGUGCAGUAGCUCC
crRNA‐2	GAAAUUAAUACGACUCACUAUAGGGUAAUUUCUACUAAGUGUAGAUGUUAGUUGUGCAGUAGCUCCUUA
ssDNA‐reporter	FAM‐TTATT‐BHQ1
qPCR‐F	CATCTATTATAGCATCAGCATCAGA
qPCR‐R	GATACTCCTCCTCGTTGGTCTAC
qPCR‐Probe	FAM‐TGTAGGCCGTGTATCAGCATCCATT‐BHQ1

Abbreviations: BHQ, black hole quencher; FAM, carboxyfluorescein; qPCR, quantitative polymerase chain reaction.

### 2.5 Preparation of crRNA

Templates for crRNA transcription (NTS and TS) were annealed in hybridization buffer (20 mM Tris–HCl, pH 7.5; 100 mM KCl; 5 mM MgCl_2_) by heating to 95°C for 5 min and cooling to room temperature (Chen et al. 2018). Following annealing, dsDNA was transcribed into crRNA using the HiScribe T7 High Yield RNA Synthesis Kit (NEB, Beijing) during a 12–16 h incubation at 37°C. CrRNA was subsequently purified via the Monarch RNA Cleanup Kit (NEB) and quantified using an Implen N60.

### 2.6 Screening of crRNA and Optimization of the CRISPR/Cas12a Detection System

A 20 μL CRISPR/Cas12a reaction system was prepared containing 150 nM Cas12a protein, 100 nM crRNA, 0.5 μM ssDNA reporter molecule (ssDNA‐reporter), 60 ng pcDNA3.1‐MPXV‐I, and 2 μL of 10 × Borealis buffer. crRNA‐I‐1 and crRNA‐I‐2 were separately added, and the system was incubated at 37°C for 40 min. Fluorescence signals were collected every minute using real‐time quantitative PCR (StepOnePlus Real‐Time Fluorescence PCR System), and fluorescence signal intensity and peak initiation time were compared among different crRNA groups.

Cas12a protein at varying concentrations (75, 112.5, 150, 187.5, 225 nM) was introduced into the CRISPR/Cas12a system. The optimal Cas12a protein concentration was determined based on fluorescence signal intensity and time‐to‐peak. Samples were subsequently supplemented with crRNA at varying concentrations (25, 50, 100, 200 nM) to screen for the optimal crRNA concentration. Finally, ssDNA‐reporter was introduced to the samples at graded concentrations (0.5, 0.75, 1, 1.25, 1.5 μM) to determine the optimal ssDNA‐reporter concentration.

### 2.7 Screening of RPA Primers

The RPA premix was prepared using the TwistAmp Basic kit (TwistDx, UK) according to the manufacturer's instructions. The RPA pellet was resuspended in 29.5 μL of rehydration buffer. After spiking with 2.5 μL of magnesium acetate and 2.4 μL of each primer (F/R, 10 μM), we brought the final volume to 45 μL with DEPC‐treated water before adding 5 μL of template. A ten‐fold serial dilution (4.37 × 10^2^–4.37 × 10^4^ copies/μL) of the recombinant plasmid pcDNA3.1‐MPXV‐I was used as the RPA template, and amplification was performed for 20 min using three distinct RPA primer pairs. The amplification products from RPA were analysed using agarose gel electrophoresis to determine the amplification efficiencies of the different primer pairs.

### 2.8 Development and Optimization of the RPA‐CRISPR/Cas12a Assay

The single‐tube RPA‐CRISPR/Cas12a assay was performed using a compartmentalized tube sleeve design. Briefly, 20 μL of the 45 μL RPA reaction mixture described above was aliquoted to the bottom of a 1.5 mL microcentrifuge tube, followed by the addition of 5 μL of nucleic acid template directly into the 20 μL RPA mixture. Secondly, the preconfigured CRISPR/Cas12a detection mixture was loaded into a capless PCR tube. Finally, the PCR tube was inverted and inserted into the 1.5 mL microcentrifuge tube as an inner sleeve. Owing to surface tension, the CRISPR/Cas12a mixture remained inside the inverted PCR tube and was physically separated from the RPA mixture during amplification. After RPA amplification, brief centrifugation was performed to release the CRISPR/Cas12a mixture into the amplified RPA reaction, followed by CRISPR/Cas12a‐mediated signal generation.

For assay optimization, recombinant plasmid pcDNA3.1‐MPXV‐I was ten‐fold serially diluted from 4.37 × 10^0^ to 4.37 × 10^4^ copies/μL and used as the template. RPA was carried out for 20 min across a temperature gradient (42°C, 39°C, 37°C). The reactions were then briefly centrifuged to merge them with the CRISPR/Cas12a system, followed by a 20 min incubation at 37°C. Results were documented by visual inspection or using smartphone‐captured images. Pixel values from the green channel of RGB images were extracted and analysed using ImageJ software, and the results were statistically evaluated by two‐way ANOVA using GraphPad Prism.

### 2.9 Evaluation of Analytical Sensitivity, Specificity, and Repeatability of the RPA‐CRISPR/Cas12a Assay

A preliminary sensitivity screening was first performed using ten‐fold serial dilutions of the recombinant plasmid ranging from 4.37 × 10^3^ to 4.37 × 10^0^ copies/μL under the optimized reaction conditions. The preliminary limit of detection was defined as the lowest concentration at which all three independent replicates yielded positive results. Subsequently, we selected five concentrations straddling 4.37 × 10^1^ copies/μL—specifically 43.70, 21.85, 10.93, 5.46, and 2.73 copies/μL. Each level was tested with 20 independent replicates to determine the LOD95. After the reaction, results were documented using smartphone‐captured images under fixed imaging conditions. Green‐channel pixel intensities were measured in ImageJ. We then fit a probit regression model in IBM SPSS Statistics to back‐calculate the LOD95.

To preliminarily evaluate the performance of the assay under room‐temperature conditions, recombinant plasmids were ten‐fold serially diluted from 4.37 × 10^3^ to 4.37 × 10^0^ copies/μL and tested using the RPA‐CRISPR/Cas12a assay at 25°C. Results were documented by visual inspection or using smartphone‐captured images. Pixel values from the green channel of RGB images were extracted and analysed using ImageJ software.

The RPA‐CRISPR/Cas12a assay was also performed using recombinant plasmids (pcDNA3.1‐MPXV‐I, pcDNA3.1‐MPXV‐II, BV, CPXV) and DNA templates (VVT, VZV) to evaluate the assay specificity. Results were documented with visual inspection or using smartphone‐captured images. Green‐channel pixel intensities were measured in ImageJ and assessed by one‐way ANOVA in GraphPad Prism.

To evaluate the repeatability of the RPA‐CRISPR/Cas12a assay, recombinant plasmids at 4.37 × 10^3^, 4.37 × 10^2^, 4.37 × 10^1^, and 4.37 × 10^0^ copies/μL were used as templates, and a negative control was included in each experiment. For intra‐assay repeatability, each concentration was tested in triplicate within the same experimental run. For inter‐assay repeatability, the same concentrations were tested in three independent runs performed on different days, with each concentration tested in triplicate in each run. Results were documented using smartphone‐captured images under fixed imaging conditions. Green‐channel pixel intensities were measured in ImageJ. The mean value, standard deviation (SD), and coefficient of variation (CV) were calculated based on pixel values.

### 2.10 Evaluation of Simulated Clinical Samples Using the RPA‐CRISPR/Cas12a Assay

To validate the applicability of the RPA‐CRISPR/Cas12a assay in actual clinical samples, total DNA was extracted from Vero cells (African green monkey kidney epithelial cells) and 293 T cells (human embryonic kidney 293 T cells). Subsequently, recombinant plasmids at 4.37 × 10^8^, 4.37 × 10^6^, 4.37 × 10^4^, 4.37 × 10^2^, 4.37 × 10^1^, and 4.37 × 10^−1^ copies/μL were spiked into the extracted total DNA. The panel comprised 32 simulated samples: 14 Vero cell and 14,293 T cell DNA backgrounds spiked with the recombinant plasmid, plus 2 negative controls for each cell line. We then tested these samples head‐to‐head using both the RPA‐CRISPR/Cas12a assay and standard qPCR. The qPCR detection was performed using the assay established by Margaret G. Mills et al., with the primers and probe listed in Table 1 (Mills et al. 2023). The interpretation of positive and negative results was based on both the presence of a typical sigmoidal amplification curve and whether a Ct value was generated.

## 3 Results

### 3.1 Preparation and Activity Validation of Cas12a Protein

Cas12a protein was analysed using SDS‐PAGE (Figure S1A). Thin‐layer scanning (TLS) confirmed the Cas12a preparation was 96.98% pure (Figure S1B). We then gauged its enzymatic activity using our established CRISPR/Cas12a platform. As expected, the assay lit up with a strong fluorescent signal in the presence of the positive control, while the negative control remained dark (Figure S1C), confirming potent trans‐cleavage activity in the purified protein.

### 3.2 Screening of crRNA

**FIGURE 2:**
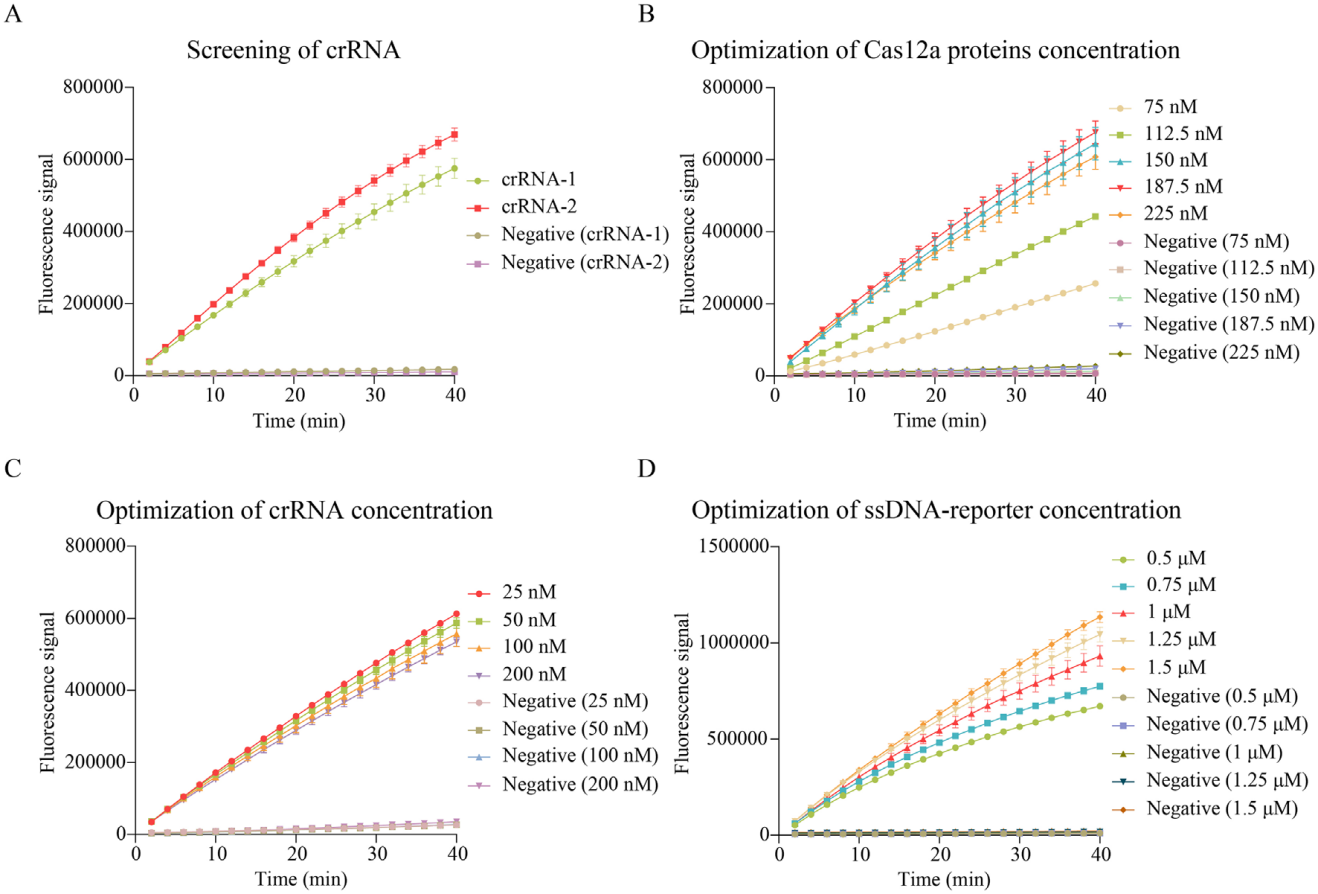
Optimization of the CRISPR/Cas12a system. (A) Screening of crRNAs with 4.37 × 10^9^ copies/μL recombinant plasmid as the target. Fluorescence intensity was monitored with real‐time quantitative PCR. (B) Detection with different concentrations of Cas12a protein. (C) Detection with different concentrations of crRNA. (D) Detection with different concentrations of ssDNA‐reporter. Error bars represent the mean ± standard deviation (SD) of three independent experiments (*n* = 3).

To enhance the sensitivity and specificity of the RPA‐CRISPR/Cas12a assay, crRNA screening was performed. Although both crRNAs specifically recognized the target sequence, they exhibited distinct fluorescence signal intensities. crRNA‐I‐2 demonstrated superior fluorescence signal intensity and was therefore used in the subsequent experiments (Figure 2A).

### 3.3 Optimization of the CRISPR/Cas12a System

To push the sensitivity of the RPA‐CRISPR/Cas assay, we first titrated the Cas12a protein. Fluorescence peaked at a final concentration of 187.5 nM (Figure 2B). With that settled, we screened crRNA levels and observed maximal signal at 25 nM (Figure 2C), effectively locking in our core reaction mix. We then turned to the ssDNA reporter, where 1 μM yielded robust fluorescence (Figure 2D). Balancing performance with cost, we adopted this 1 μM concentration for all subsequent experiments.

### 3.4 Screening of RPA Primers and Optimization of Amplification Temperature

**FIGURE 3:**
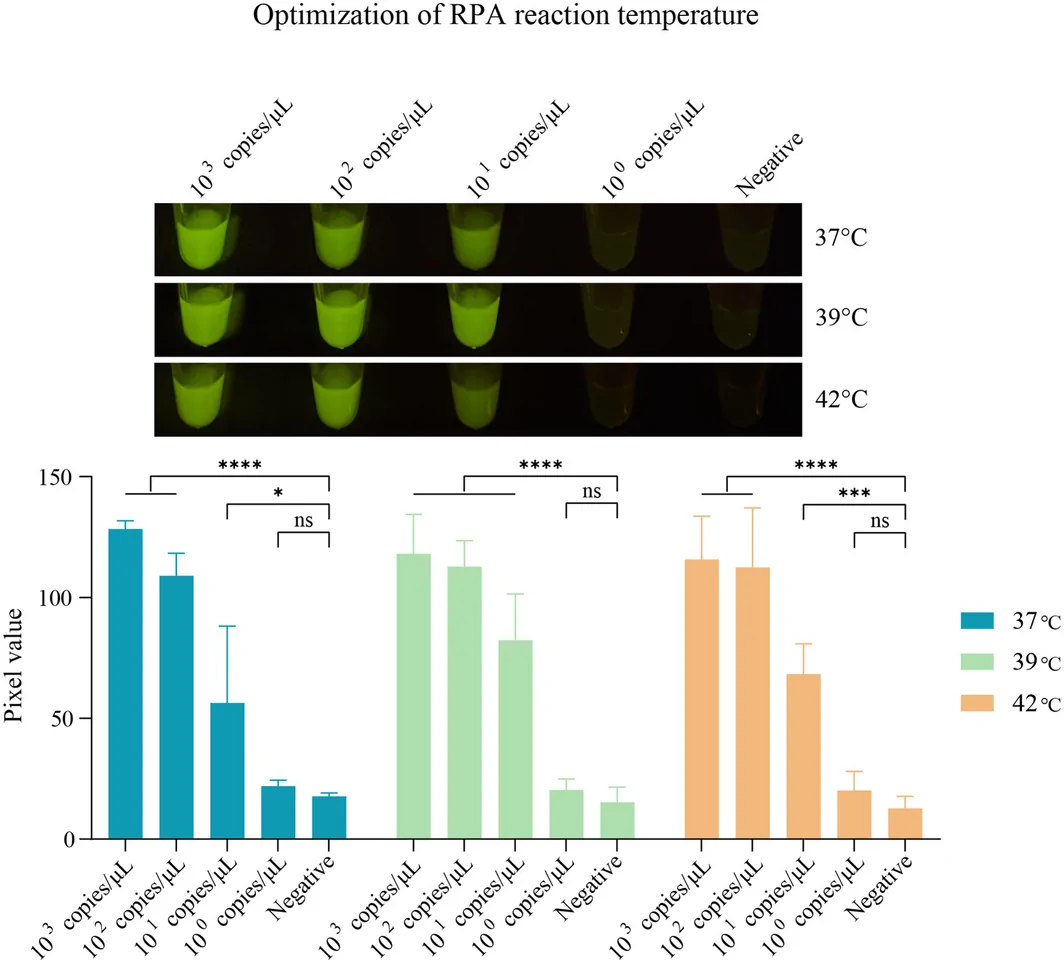
Optimization of reaction conditions for the RPA‐CRISPR/Cas12a assay. The optimal amplification temperature was determined by testing 10‐fold serially diluted recombinant plasmid (4.37 × 10^0^ copies/μL to 4.37 × 10^3^ copies/μL). Images were captured using an iPhone 12 Pro Max, and pixel values of the green channel were extracted using ImageJ software. Data were analysed using two‐way ANOVA; error bars represent mean ± SD (*n* = 3). Statistical analysis was performed using GraphPad Prism, with ns indicating no significant difference (*p* > 0.05); *p < 0.05; ***p < 0.001; ****p < 0.0001.

As shown in Figure 1, the assay was performed in a closed, compartmentalized tube system consisting of a 1.5 mL microcentrifuge tube and an inverted capless PCR tube, which allowed the RPA and CRISPR/Cas12a reactions to be separated during amplification and mixed by centrifugation before signal detection. To improve the amplification efficiency of the RPA reaction, RPA primers were screened. The results demonstrated that the primer pair RPA‐3‐F/R exhibited the highest amplification efficiency and was therefore selected for subsequent experiments (Figure S2). Additionally, to enhance the sensitivity of the RPA‐CRISPR/Cas12a assay, the RPA amplification temperature was optimized. The assay successfully detected recombinant plasmid at 4.37 × 10^1^ copies/μL at 39°C, and since the overall fluorescence signal was stronger at 39°C, this temperature was determined to be the optimal amplification temperature for the RPA‐CRISPR/Cas12a assay (Figure 3).

### 3.5 Evaluation of Analytical Sensitivity and Specificity of the RPA‐CRISPR/Cas12a Assay

**FIGURE 4:**
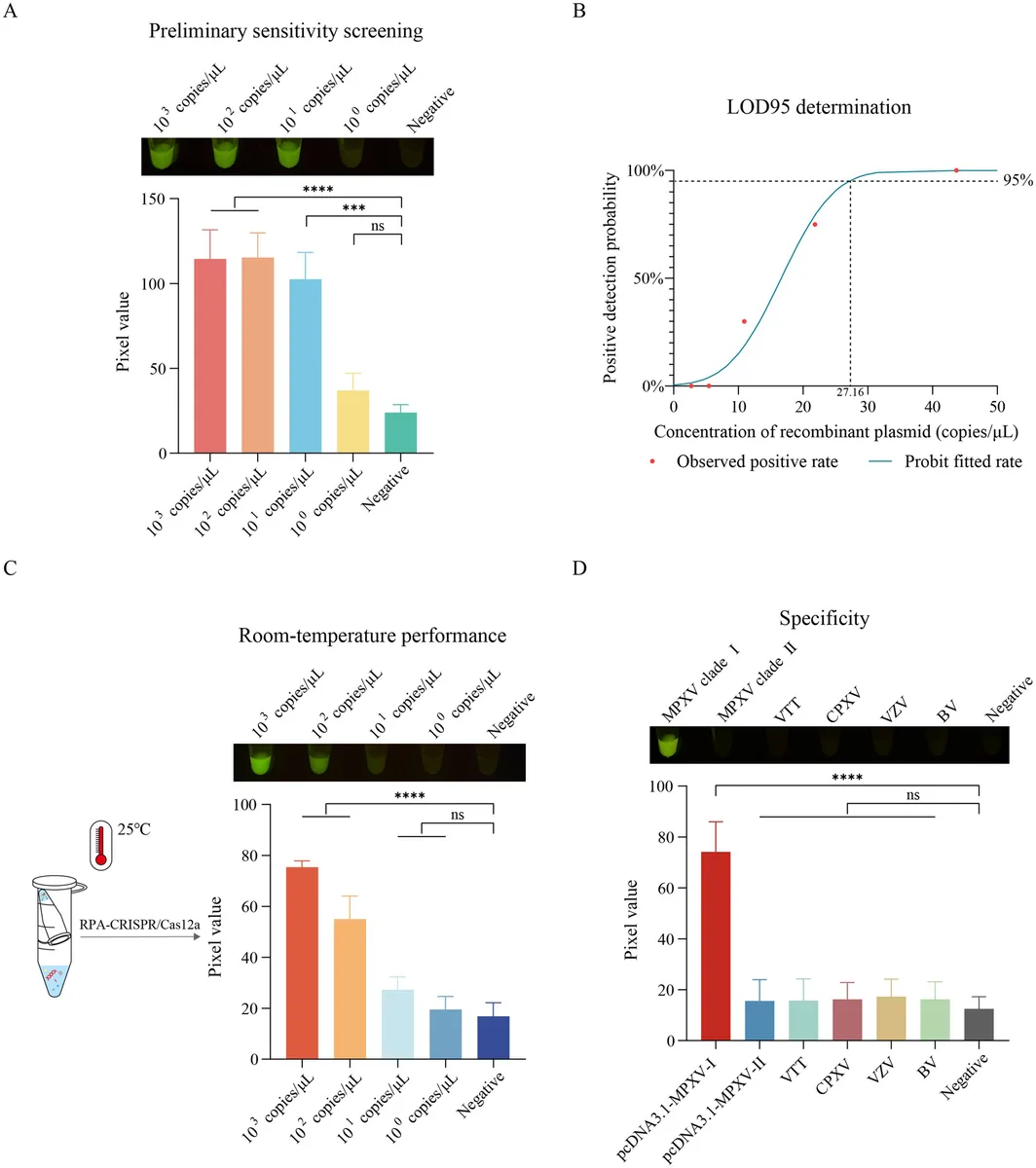
Evaluation of analytical sensitivity, room‐temperature performance, and specificity of the RPA‐CRISPR/Cas12a assay. (A) Preliminary sensitivity screening using 10‐fold serially diluted recombinant plasmids under the optimized reaction conditions. (B) Probit regression analysis for LOD95 determination. Solid points represent the observed positive detection rates at each recombinant plasmid concentration, and the fitted curve represents the probit regression model. The horizontal dashed line indicates the 95% detection probability, and the vertical dashed line indicates the estimated LOD95. (C) Preliminary evaluation of assay performance at room temperature (25°C) using 10‐fold serially diluted recombinant plasmids. (D) Specificity evaluation of the assay using MPXV clade I, MPXV clade II, VVT, CPXV, VZV, BV, and the negative control. Images were captured with an iPhone 12 Pro Max, and pixel values of the green channel were extracted using ImageJ software. For panels A, C, and D, data were analysed using one‐way ANOVA; error bars represent the mean ± SD of three independent experiments (*n* = 3). Statistical analysis was performed using GraphPad Prism: Ns, not significant (*p* > 0.05); ***p < 0.001; ****p < 0.0001.

Preliminary sensitivity screening using ten‐fold serial dilutions showed that the RPA‐CRISPR/Cas12a assay could detect recombinant plasmids down to 4.37 × 10^1^ copies/μL under the optimized reaction conditions, while no positive signal was observed at 4.37 × 10^0^ copies/μL (Figure 4A). Based on this result, five concentrations around the preliminary detection limit were selected for LOD95 analysis. The assay picked up all 20 replicates at 43.70 copies/μL and 15/20 at 21.85 copies/μL, with detection dropping to 6/20 at 10.93 copies/μL and nil at lower concentrations (Table 2). Probit regression placed the LOD95 at 27.16 copies/μL, with a 95% confidence interval of 23.06–35.24 copies/μL (Figure 4B).

**TABLE 2:** Limit of detection of the RPA‐CRISPR/Cas12a assay.

Concentration (copies/μL)	Positive/total replicates	Positive detection rate
43.70	20/20	100%
21.85	15/20	75%
10.93	6/20	30%
5.46	0/20	0%
2.73	0/20	0%

We next took a preliminary look at how the assay holds up at ambient temperature. At 25°C, it reliably detected the plasmid at 4.37 × 10^2^ copies/μL, though signal strength attenuated at lower template concentrations (Figure 4C). While this doesn't match the sensitivity of our optimized thermostatted conditions, it confirms the assay remains viable in field settings where incubators aren't an option.

Specificity was then assessed using a panel of recombinant plasmids (pcDNA3.1‐MPXV‐I, pcDNA3.1‐MPXV‐II, BV, CPXV) and viral DNA (VVT, VZV). Robust fluorescence was exclusive to the clade I plasmid, with no detectable signal from heterologous viruses or the no‐template control (Figure 4D).

### 3.6 Intra‐Assay and Inter‐Assay Repeatability of the RPA‐CRISPR/Cas12a Assay

To evaluate the stability of the RPA‐CRISPR/Cas12a assay, intra‐assay and inter‐assay repeatability were assessed using recombinant plasmids at 4.37 × 10^3^, 4.37 × 10^2^, 4.37 × 10^1^, and 4.37 × 10^0^ copies/μL. In the intra‐assay repeatability, the CV values of the assay ranged from 1.032% to 8.509%. In the inter‐assay repeatability test, which was performed in three independent runs on different days, the CV values of the assay ranged from 2.279% to 8.483% (Table 3). The negative controls remained negative throughout the experiments. These results demonstrated good intra‐assay and inter‐assay repeatability of the established RPA‐CRISPR/Cas12a assay.

**TABLE 3:** Repeatability of the RPA‐CRISPR/Cas12a assay.

Concentration (copies/μL)	Intra‐assay mean ± SD	Intra‐assay CV (%)	Inter‐assay mean ± SD	Inter‐assay CV (%)
4.37 × 10^3^	129.624 ± 1.338	1.032	128.044 ± 2.918	2.279
4.37 × 10^2^	128.046 ± 7.741	6.045	128.331 ± 4.738	3.692
4.37 × 10^1^	120.236 ± 7.468	6.211	124.535 ± 4.609	3.701
4.37 × 10^0^	21.519 ± 1.831	8.509	32.938 ± 2.794	8.483

### 3.7 Evaluation of the RPA‐CRISPR/Cas12a Assay With Simulated Clinical Samples

**FIGURE 5:**
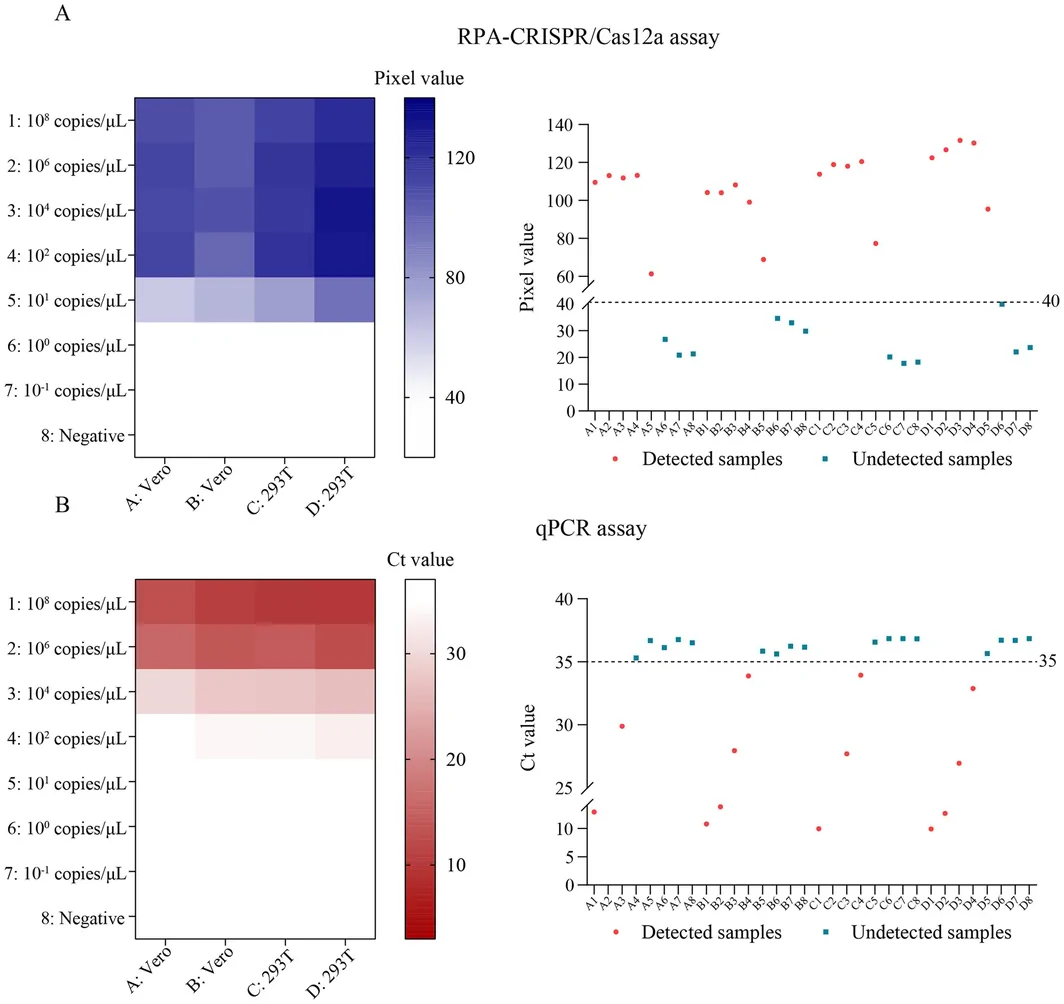
Evaluation of the RPA‐CRISPR/Cas12a assay in simulated clinical experiments. (A) Detection of 32 simulated clinical samples (28 positive and 4 negative samples) using the RPA‐CRISPR/Cas12a assay, with each sample tested in three technical replicates. Images were captured with an iPhone 12 Pro Max, and green channel pixel values were extracted using ImageJ software. Data were statistically analysed using GraphPad Prism. A pixel value > 40 was defined as a positive result. (B) Detection of 32 simulated clinical samples (28 positive and 4 negative samples) using qPCR, with each sample tested in three technical replicates. A *C*_t_ < 35 with a typical sigmoidal amplification curve was defined as a positive result.

We prepared simulated samples by spiking different concentrations of recombinant plasmids into total DNA extracted from Vero cells and 293 T cells. For the RPA‐CRISPR/Cas12a assay, green‐channel pixel intensities were extracted from smartphone‐captured images using ImageJ under fixed imaging conditions. Based on the signal distribution of negative controls and dilution‐series simulated samples, a pixel value > 40 was defined as positive, whereas a pixel value ≤ 40 was defined as negative. This threshold was used to distinguish true fluorescence signals from background fluorescence in the simulated sample evaluation. For qPCR analysis, some negative‐control samples showed only weak late upward fluorescence drift after 35 cycles, rather than typical sigmoidal amplification curves. Therefore, these late non‐sigmoidal weak signals were interpreted as negative. Based on this observation, samples with a typical sigmoidal amplification curve and *C*_t_ < 35 were considered positive, whereas samples with *C*_t_ ≥ 35 and no typical sigmoidal amplification curve, or no fluorescence signal, were interpreted as negative. Using the RPA‐CRISPR/Cas12a assay, 10 of 14 spiked samples were detected in both the Vero cell and 293 T cell DNA backgrounds (Figure 5A and Table 4). In comparison, qPCR detected 7 of 14 spiked samples in the Vero cell DNA background and 8 of 14 samples in the 293 T cell DNA background (Figure 5B and Table 5). These results indicate that, under the simulated sample conditions, the RPA‐CRISPR/Cas12a assay detected more low‐concentration spiked samples than qPCR.

**TABLE 4:** Detection of simulated clinical samples by the RPA‐CRISPR/Cas12a assay.

Concentration (copies/μL)	Pixel value			
	A: Vero	B: Vero	C: 293 T	D: 293 T
1: 10^8^ copies/μL	109.525 ± 32.757	104.185 ± 13.025	113.784 ± 20.629	122.504 ± 9.194
2: 10^6^ copies/μL	113.051 ± 35.446	104.079 ± 13.105	118.932 ± 21.923	126.628 ± 8.054
3: 10^4^ copies/μL	111.773 ± 35.414	108.172 ± 11.691	118.031 ± 24.285	131.665 ± 9.085
4: 10^2^ copies/μL	113.160 ± 32.161	99.127 ± 16.227	120.444 ± 21.492	130.290 ± 15.822
5: 10^1^ copies/μL	61.250 ± 16.924	68.875 ± 19.951	77.261 ± 10.958	95.446 ± 22.867
6: 10^0^ copies/μL	26.726 ± 15.171	34.561 ± 17.365	20.163 ± 1.456	39.836 ± 15.095
7: 10^−1^ copies/μL	20.839 ± 10.322	32.857 ± 10.023	17.756 ± 2.521	22.022 ± 3.381
8: Negative	21.245 ± 10.913	29.729 ± 4.974	18.221 ± 2.095	23.646 ± 3.349

**TABLE 5:** Detection of simulated clinical samples by qPCR.

Concentration (copies/μL)	Ct value			
	A: Vero	B: Vero	C: 293 T	D: 293 T
1: 10^8^ copies/μL	12.896 ± 0.088	10.756 ± 0.198	9.919 ± 0.138	9.879 ± 0.074
2: 10^6^ copies/μL	15.880 ± 0.086	13.818 ± 0.155	14.328 ± 0.338	12.642 ± 0.276
3: 10^4^ copies/μL	29.878 ± 0.116	27.937 ± 0.074	27.684 ± 0.308	26.942 ± 0.033
4: 10^2^ copies/μL	35.294 ± 0.420	33.865 ± 0.081	33.928 ± 0.051	32.872 ± 0.128
5: 10^1^ copies/μL	36.670 ± 0.496	35.843 ± 0.181	36.546 ± 0.725	35.640 ± 0.296
6: 10^0^ copies/μL	36.123 ± 0.806	35.622 ± 0.551	36.830 ± 0.251	36.710 ± 0.277
7: 10^−1^ copies/μL	36.755 ± 0.303	36.237 ± 0.601	36.841 ± 0.208	36.691 ± 0.257
8: Negative	36.503 ± 0.466	36.156 ± 0.552	36.820 ± 0.361	36.829 ± 0.333

## 4 Discussion

Mpox is a highly transmissible zoonotic disease posing a significant risk to public health (Falendysz et al. 2023). According to WHO data, 52,845 reported confirmed Mpox cases, including 215 deaths among confirmed cases, were reported globally from 1 January to 31 December 2025. In 2024, the second major outbreak of Mpox emerged globally, with most cases identified as originating from a novel sublineage Ib of MPXV clade I, exhibiting enhanced virulence and transmissibility (Shete et al. 2024; Kumar et al. 2025). On August 14, 2024, WHO declared the ongoing Mpox outbreak a Public Health Emergency of International Concern. The sublineage Ib is associated with more severe clinical manifestations. Early and accurate diagnosis enables timely medical intervention, effectively mitigating MPXV‐induced tissue damage and reducing residual scar formation (Srivastava et al. 2024). Therefore, rapid detection technologies targeting MPXV clade I are of critical importance for clinical treatment, epidemic control, and epidemiological investigations.

This study established a one‐step visual detection assay targeting MPXV clade I by integrating RPA with the CRISPR/Cas12a system. To reduce cross‐contamination risks associated with tube uncapping and sample transfer, we developed a streamlined single‐tube strategy that physically integrates nucleic acid amplification and CRISPR‐based detection while keeping the two reactions separated before signal detection (Xiong et al. 2020; Cho et al. 2024). Previous studies have shown that direct premixing of isothermal amplification reagents with CRISPR/Cas components may reduce assay sensitivity because amplification and Cas‐mediated cleavage reactions are not fully compatible in a homogeneous one‐pot system. In particular, premature activation of Cas12a during amplification may consume newly generated amplicons or target templates, thereby limiting amplification efficiency (Hu et al. 2022; Cheng et al. 2025; Nalefski et al. 2025). To address this issue, we spatially segregated the RPA reagents and CRISPR/Cas12a detection reagents using a compartmentalized tube sleeve design. After 20 min of amplification, centrifugation triggered reagent mixing without tube uncapping, allowing CRISPR/Cas12a‐based signal generation while minimizing aerosol contamination.

In the simulated sample evaluation, the RPA‐CRISPR/Cas12a assay detected 10 of 14 plasmid‐spiked samples in the Vero cell DNA background and 10 of 14 samples in the 293 T cell DNA background. The detected samples contained recombinant plasmids at or above 4.37 × 10^1^ copies/μL, which was within the detectable range of the assay. In contrast, the undetected spiked samples contained target concentrations below this level. Therefore, these negative results were mainly attributable to target concentrations below the analytical detection limit of the assay rather than random false‐negative results or poor assay reproducibility. By comparison, qPCR detected 7 of 14 spiked samples in the Vero cell DNA background and 8 of 14 samples in the 293 T cell DNA background. The qPCR‐detected samples were mainly those containing recombinant plasmids at or above 4.37 × 10^2^ copies/μL, whereas the undetected samples were mostly low‐copy samples at or below 4.37 × 10^1^ copies/μL. These results indicate that the detection outcomes of both methods were concentration‐dependent, and the negative results were primarily associated with low target concentrations rather than assay instability.

The assay developed in this study was designed as a rapid and specific tool for MPXV clade I detection. This focus is clinically and epidemiologically meaningful because MPXV clade I has been reported to be associated with greater virulence than clade II. Therefore, rapid identification of MPXV clade I is important for clinical risk assessment, outbreak investigation, and surveillance of circulating MPXV clades. In clinical or field settings, suspected cases should first be evaluated together with clinical symptoms, exposure history, and the currently circulating MPXV clades in the relevant region. For diagnostic confirmation, a commercial MPXV detection kit or a validated universal MPXV assay should first be used to confirm MPXV infection. MPXV‐positive samples could then be further analysed using clade I‐ and clade II‐specific assays. In addition, our laboratory previously established a visual nucleic acid assay for MPXV that enables universal MPXV detection and clade II differentiation (Huang et al. 2023). The present assay complements this previous work by providing a clade I‐specific detection component, thereby supporting more efficient discrimination between MPXV clades I and II within an integrated MPXV detection strategy.

Several CRISPR/Cas‐based assays have been reported for MPXV detection, but their assay formats and application settings differ from the present study. Gong et al. developed an RPA‐CRISPR/Cas12a assay that could differentiate the Central Africa and West Africa clades of MPXV, with a detection limit of 5–10 copies/reaction; however, the assay required separate amplification and CRISPR detection steps with fluorescence‐based readout (Gong et al. 2023). Ahamed et al. reported an RPA‐SCAN platform with an LOD of 16 copies/μL at a 95% confidence level, comparable to the LOD95 of the present assay, but requiring nanopore‐based detection (Ahamed et al. 2024). Guo et al. established a one‐step LAMP‐CRISPR/Cas12b assay for visual MPXV detection within 40 min, although it was not designed as a clade I‐specific assay (Guo et al. 2025). In comparison, the present assay is characterized by clade I specificity, a one‐pot RPA‐CRISPR/Cas12a, visual readout, and low equipment requirements.

However, current crRNA design limitations stem from restrictive protospacer adjacent motif (PAM) sequences and from the high sequence homology (96.8%) between MPXV clades I and II (Dronina et al. 2022; Schuele et al. 2024). Nevertheless, crRNA design in CRISPR/Cas13 and Cas14 systems circumvents protospacer adjacent motif (PAM) sequence constraints, significantly broadening the crRNA selection scope (Hillary and Ceasar 2023). Therefore, future studies should prioritize the optimization of CRISPR/Cas system selection. Another limitation of this study is that the analytical evaluation was mainly performed using recombinant plasmids. Although simulated clinical samples containing cellular DNA backgrounds were used to preliminarily assess the applicability of the assay, this strategy cannot fully reproduce the complete workflow of viral nucleic acid extraction from clinical specimens. In particular, plasmid‐based templates may not fully reflect the effects of viral particle lysis, nucleic acid extraction efficiency, and complex clinical sample matrices on assay performance. Due to the unavailability of MPXV clade I clinical samples and the biosafety requirements associated with infectious MPXV, validation using real virus was not performed in this study. Therefore, further evaluation using authentic clinical specimens or pseudovirus‐based samples will be necessary to fully assess the clinical performance of this assay.

## 5 Conclusion

In conclusion, this study established a rapid and visual one‐pot RPA‐CRISPR/Cas12a assay for the specific detection of MPXV clade I. The assay can be completed within 40 min and achieved a LOD95 of 27.16 copies/μL under the optimized reaction conditions. Specificity testing revealed no cross‐reactivity with MPXV clade II or related viruses. The assay exhibited high precision, with low variability across intra‐ and inter‐assay runs. Detection capability was maintained at ambient temperature (25°C), suggesting field‐deployable potential. In simulated samples, the assay outperformed standard qPCR in positive detection rates, indicating strong utility for clade I surveillance. With a simple workflow, visual readout, and low equipment requirement, this assay provides a practical tool for rapid and decentralized detection of MPXV clade I.

## Author Contributions

**Boyi Li:** data curation, investigation, methodology, writing – original draft. **Luyao Liu:** validation. **Kaikai Jin:** formal analysis, investigation, methodology. **Zanheng Huang:** formal analysis, investigation. **Tianyi Zhang:** validation. **Rong Gao:** data curation. **Huanxin Chen:** validation. **Lijuan Niu:** formal analysis. **Changqi Fan:** formal analysis. **Haili Zhang:** writing – review and editing. **Pei Huang:** project administration, writing – review and editing. **Hualei Wang:** methodology, project administration, writing – review and editing.

## Funding

This work was supported by National Key Research and Development Program of China (2021YFF0703600). The science and technology development program of Jilin Province (20250203114SF).

## Ethics Statement

The authors have nothing to report.

## Conflicts of Interest

The authors declare no conflicts of interest.

## Supporting information


**Figure S1:** Preparation and activity validation of Cas12a protein. (A) The collected flow‐through and elution fractions from the protein purification process along with the purified protein were analysed by SDS‐PAGE. (B) The purity of Cas12a protein was analysed by TLS. (C) The activity of Cas12a protein was validated using the confirmed positive CRISPRS/Cas12a system.
**Figure S2:** Screening of RPA primers. The recombinant plasmid pcDNA3.1‐MPXV‐I with 10‐fold serial dilutions (4.37 × 102–4.37 × 104 copies/μL) was used as template for RPA reactions. The amplification products were analysed by agarose gel electrophoresis to determine the amplification efficiencies of the primer pairs.

## Data Availability

The data that support the findings of this study are available from the corresponding author upon reasonable request.
