# Supplementary material for: One‐Pot RPA‐CRISPR/Cas12a Assay With Visual Readout for the Ultra‐Specific Detection of Monkeypox Virus Clade I
Source: Microb Biotechnol. 2026 Sep 6;19(9):e70437. doi: 10.1111/1751-7915.70437 (PMC13547839; doi:10.1111/1751-7915.70437)
Supplement: Supplementary file 1 [file MBT2-19-e70437-s001.docx]

**Supporting information**

Boyi Li^1^, Luyao Liu^1^, Kaikai Jin^1^, Zanheng Huang^1^, Tianyi Zhang^1^, Rong Gao^1^, Huanxin Chen^1^, Lijuan Niu^1^, Changqi Fan^2^, Haili Zhang^1^, Pei Huang^1*^, Hualei Wang^1*^

1 State Key Laboratory for Diagnosis and Treatment of Severe Zoonotic Infectious Diseases, Key Laboratory for Zoonosis Research of the Ministry of Education, Institute of Zoonosis, College of Veterinary Medicine, Jilin University, Jilin 130062, China

2 Zhili College, Tsinghua University, Beijing, 100084, China

^*^ Corresponding author

E-mail: wanghualei@jlu.edu.cn (HW)

E-mail: huangpei@jlu.edu.cn (PH)

**Figure S1. Preparation** **and activity validation of Cas12a protein.** (A) The collected flow-through and elution fractions from the protein purification process along with the purified protein were analyzed by SDS-PAGE. (B) The purity of Cas12a protein was analyzed by TLS. (C) The activity of Cas12a protein was validated using the confirmed positive CRISPRS/Cas12a system.

**Figure S2. Screening of RPA primers.** The recombinant plasmid pcDNA3.1-MPXV-I with ten-fold serial dilutions (4.37×10^2^ to 4.37×10^4^ copies/μL) was used as template for RPA reactions. The amplification products were analyzed by agarose gel electrophoresis to determine the amplification efficiencies of the primer pairs.
